# Switched-capacitor realization of presynaptic short-term-plasticity and stop-learning synapses in 28 nm CMOS

**DOI:** 10.3389/fnins.2015.00010

**Published:** 2015-02-02

**Authors:** Marko Noack, Johannes Partzsch, Christian G. Mayr, Stefan Hänzsche, Stefan Scholze, Sebastian Höppner, Georg Ellguth, Rene Schüffny

**Affiliations:** ^1^Chair of Highly-Parallel VLSI-Systems and Neuromorphic Circuits, Technische Universität DresdenDresden, Germany; ^2^Institute of Neuroinformatics, University of Zurich and ETH ZurichZurich, Switzerland

**Keywords:** switched-capacitor neuromorphic, stop-learning synapse, dynamic synapse, deep-submicron neuromorphic, low-leakage switched-capacitor circuits

## Abstract

Synaptic dynamics, such as long- and short-term plasticity, play an important role in the complexity and biological realism achievable when running neural networks on a neuromorphic IC. For example, they endow the IC with an ability to adapt and learn from its environment. In order to achieve the millisecond to second time constants required for these synaptic dynamics, analog subthreshold circuits are usually employed. However, due to process variation and leakage problems, it is almost impossible to port these types of circuits to modern sub-100nm technologies. In contrast, we present a neuromorphic system in a 28 nm CMOS process that employs switched capacitor (SC) circuits to implement 128 short term plasticity presynapses as well as 8192 stop-learning synapses. The neuromorphic system consumes an area of 0.36 mm^2^ and runs at a power consumption of 1.9 mW. The circuit makes use of a technique for minimizing leakage effects allowing for real-time operation with time constants up to several seconds. Since we rely on SC techniques for all calculations, the system is composed of only generic mixed-signal building blocks. These generic building blocks make the system easy to port between technologies and the large digital circuit part inherent in an SC system benefits fully from technology scaling.

## 1. Introduction

Biological synapses employ a range of plasticity mechanisms in modulating their stimulus transmission. For example short-term plasticity on the timescale of hundreds of milliseconds has been identified as a crucial constituent of dynamic neural information processing, allowing for temporal filtering (Grande and Spain, 2005), selective information transmission (Mayr et al., 2009) and pattern classification in attractor networks (Mejias and Torres, 2009). Long-term plasticity, with induction on the minute to hour scale, is used for pattern learning (Brader et al., 2007) and topology formation, allowing a network to be structured for solving a particular problem (Rubinov et al., 2011). Both of these mechanisms employ exponential time windows with time constants on the order of 10–1000 ms.

Most analog neuromorphic implementations of plasticity rely on subthreshold circuits (Indiveri et al., 2006) to achieve the small currents necessary for these long time constants. However, these are hard to port to advanced CMOS techologies, since leakage currents rapidly increase with down-scaling, reaching the range of the desired signal currents (Roy et al., 2003). Some plasticity circuits have also been implemented in OTA-C architectures (Koickal et al., 2007; Noack et al., 2011), but these suffer from the same problems with small currents. Digital plasticity circuits (Cassidy et al., 2011) are not subject to this limitation, but have limited biological veracity due to their digital state variables. For subthreshold circuits, an additional problem is the increase of device mismatch and process variation (Kinget, 2005), making transistors almost unusable for the exponential computation that subthreshold circuits rely upon. This is why even recent subthreshold neuromorphic systems have been manufactured in quite large technologies (Bartolozzi and Indiveri, 2007; Indiveri et al., 2010; Moradi and Indiveri, 2013), with the sole exception a recent design in 90 nm (Park et al., 2014).

The SC technique offers a viable alternative, as it utilizes robust charge-based signal transmission. That is, it computes with charges that are equivalent to accumulating the continuous signal currents of subthreshold circuits across time, thereby raising signal levels compared to the subthreshold approach. This approach has already been successfully applied to neuromorphic neuron implementations (Vogelstein et al., 2007; Folowosele et al., 2009).

In Mayr et al. (in press), a neuromorphic system using SC circuits has been presented that achieves biological real time operation in a 28 nm CMOS process. While (Mayr et al., in press) presents the static neuromorphic components (weight implementation, neurons, etc.) and the overall system integration, in this companion paper we focus on neuronal dynamics. Specifically, this paper presents the SC circuits that implement presynaptic adaptation and synaptic plasticity. The short-term (presynaptic) plasticity has been adapted for SC (Noack et al., 2012) from the biology-derived neurotransmitter release model of Markram et al. (1998). The long-term (synaptic) plasticity circuit implements the stop learning stochastic synapse model of Brader et al. (2007). To the best of our knowledge, this represents the first time the well-known stop-learning paradigm has been translated to SC circuits.

Vogelstein et al. (2007) and Folowosele et al. (2009) have chosen a straightforward SC approach with conventional CMOS switches, as leakage currents were not a concern in their chosen technology nodes. However, this approach is not possible in deep-submicron technologies such as the employed 28 nm process. The leakage for open switches would preclude storing a signal on the required 10–1000 ms timescale. Thus, we describe circuit techniques to reduce leakage currents, in turn allowing us to achieve high time constants. The entire neuromorphic system consists of standard analog building blocks and synthesizable digital logic, making it easy to port between technologies. As detailed later, the system architecture has been optimized for mismatch reduction.

## 2. Materials and methods

### 2.1. Overall system

**Figure 1:**
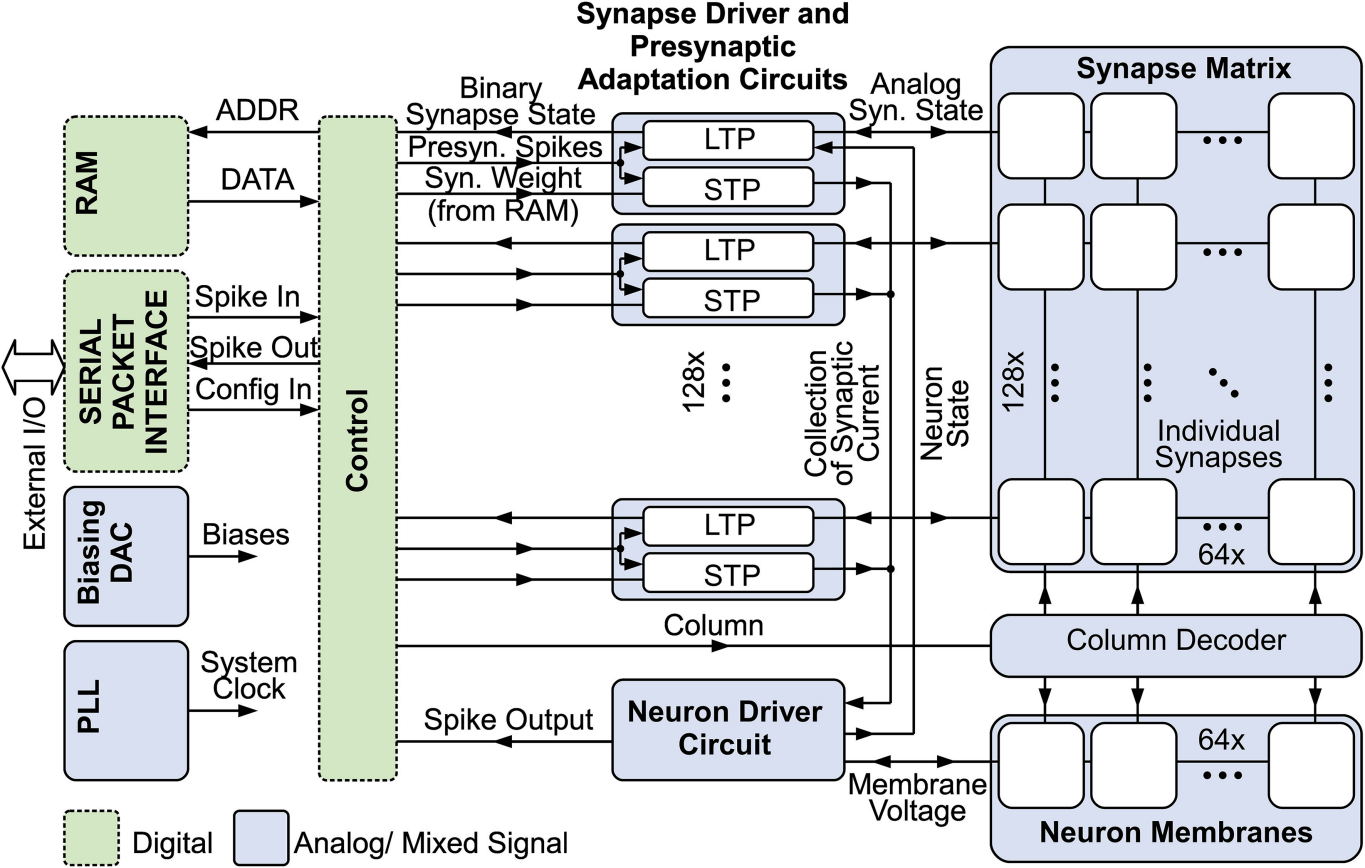
**Overview of the neuromorphic system with mixed signal SC blocks (e.g., presynaptic adaptation, synapse matrix and neurons), digital control, synaptic weight RAM, biasing DAC, PLL clock input and serial packet IO (Mayr et al., in press)**.

Figure 1 gives an overview of the system (Mayr et al., in press). 128 input circuits at the left side realize presynaptic short-term dynamics for their respective row in the synaptic matrix (Noack et al., 2012), while the 64 neurons at the bottom are driven by their respective column, providing the output (i.e., stimulation) signal as a function of the 8192 synapses in the system, which couple presynaptic input to neurons. Synaptic weights are stored in a dedicated RAM block separate from the synapse matrix.

The entire driving circuitry of presynapses, synapses and neurons is situated at the left hand side of the matrix. A state machine cycles through the columns of the synaptic matrix. At the start of the cycle, the input pulses that were registered during the last cycle are forwarded to the driver circuits and the corresponding presynaptic adaptation state is computed. Then, each synaptic column is activated sequentially, and the synaptic plasticity change of a synapse at a specific row is computed based on presynaptic pulse activity of that row and the membrane state of the neuron of the current column. Concurrently, the presynaptic pulses are integrated on the neuron. Sharing the active driver circuitry for all neurons respectively for all synapses of a row inherently reduces mismatch effects, as the only remaining mismatch between synapses is the mismatch of their state-holding capacitors. Mismatch between transistors, i.e., between active circuits, is only felt between rows.

The circuit design utilizes only digital core devices of the 28 nm SLP (super low power) technology. In contrast to the current biasing usually employed in neuromorphic ICs (Yang et al., 2012), the neuromorphic SC circuits require voltages provided by a digital-to-analog converter (DAC) to set amplitude parameters such as scaling of presynaptic adaptation, etc. This saves pins and offers an easy and robust configurability.

Time constants are set via counters that govern the switching cycles of the SC circuits. Thus, scaling of the clock frequency effectively scales the speed of the system, keeping the resolution relative to the chosen time base. As the clock speed scaling retains the relative speed of all processes, the same configuration for all parameters (amplitudes and time constants) can be used irrespective of the speed-up, nominally giving the same results. The neuromorphic system was designed for speeds from biological real-time (corresponding to a 0.62 ms full cycle time of the synaptic matrix) up to an acceleration of 100.

**Figure 2:**
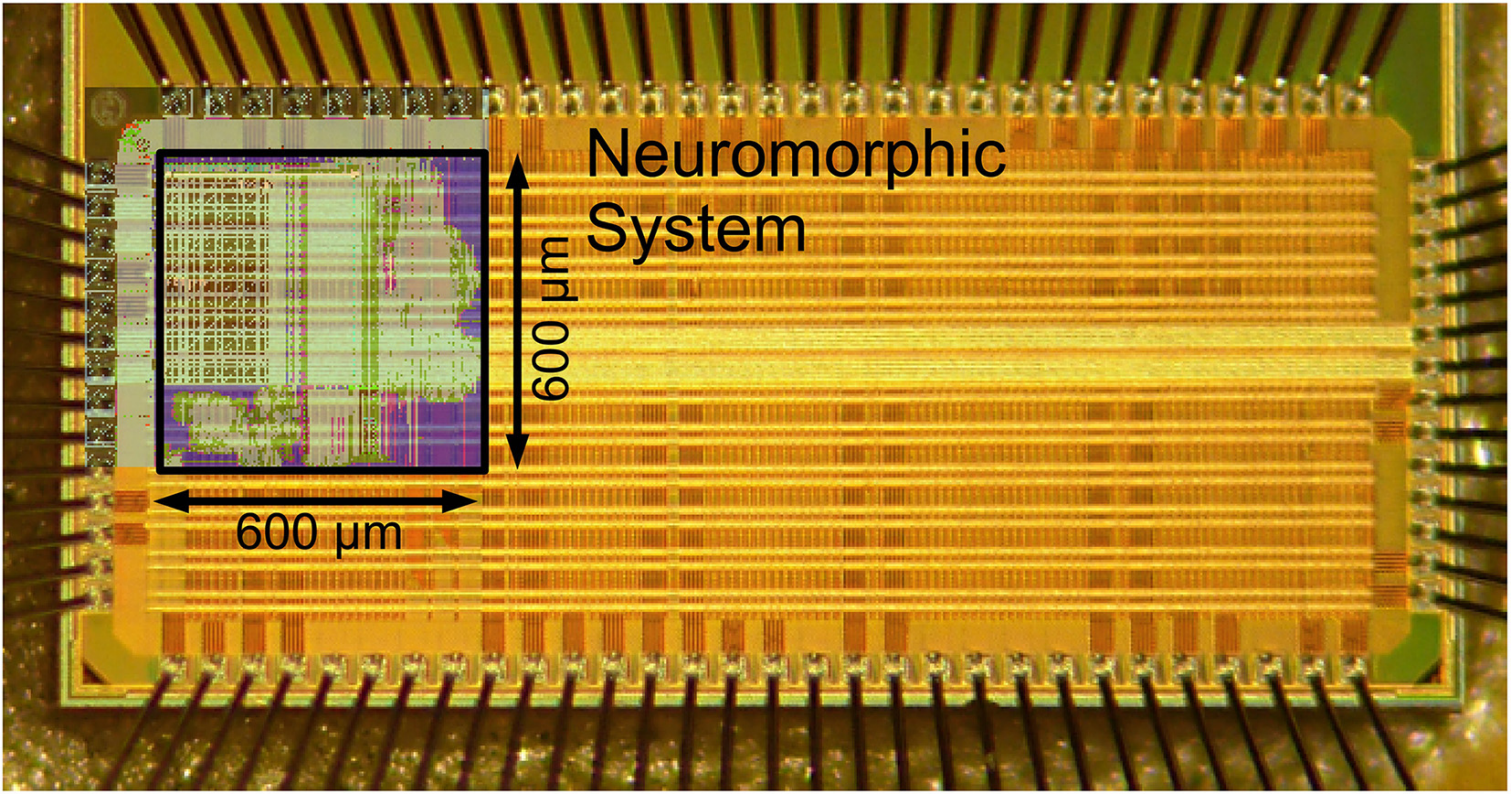
**Chip photograph with overlay of the 600 μm × 600 μm neuromorphic system layout**. Die size is 1.5 mm × 3 mm (Mayr et al., in press).

Communication with the system is provided by a JTAG interface, implementing a generic packet-based protocol. Similar to the communication setup in Hartmann et al. (2010); Scholze et al. (2011), these packets contain configuration and incoming/outgoing pulse communication data. Additionally, two configurable test outputs allow for monitoring analog voltages, such as membrane potentials. With its minimal interface, using only 6 signal pins and two bias pins (one bias current and one pin for common mode voltage), the neuromorphic system can be easily integrated into a multi-core system mediated by an FPGA. A chip photograph is shown in Figure 2. The neuromorphic system occupies 0.36 mm^2^ and is surrounded by various test structures. The overall IC has a size of 1.5 mm × 3 mm.

### 2.2. Implementation of presynaptic short-term plasticity

#### 2.2.1. Model

The presynaptic adaptation circuit implements the model of synaptic dynamics proposed in Noack et al. (2012), which is derived from a model based on biological measurements (Markram et al., 1998). The major drawback of the original approach in Markram et al. (1998) with respect to a switched-capacitor implementation is the need for a wide-range voltage multiplier for calculating the product of the facilitation and depression state variables. Existing multipliers are rather complex, very area consuming (Hong and Melchior, 1984) or need large operational amplifiers driving resistive loads (Khachab and Ismail, 1991). In contrast, the model proposed in Noack et al. (2012) is capable of approximately reproducing the original model without any multiplier circuit and with a minimum effort on analog circuitry in general.

The iterative description of the proposed model is shown in Equations (1–3):

(1) $$\;u_{n+1}=u_{n}\cdot (1-U)\cdot {\text{e}}^{-\frac{\Delta t_{n}}{\tau_{u}}}+U$$

(2) $$\;R_{n+1}=\left((1-\alpha )\cdot R_{n}+\alpha \cdot u_{n}\right)\cdot {\text{e}}^{-\frac{\Delta t_{n}}{\tau_{R}}}$$

(3) $$PSC_{n}=A\cdot (u_{n}-R_{n}).$$

It provides the amplitude *PSC_n_* of the postsynaptic current for successive presynaptic spikes incorporating their spiking history, where *n* is the number of the observed spike and Δ*t_n_* denotes the time between *n*-th and (*n* + 1)-th spike. The model is capable of reproducing facilitation and depression as well as various combinations of both mechanisms. Facilitation is modeled by variable *u*, which is adopted from Markram et al. (1998). At each incoming presynaptic spike *u* is increased by a certain amount, depending on *U*. Between spikes it exponentially decays back to *U* with time constant τ_*u*_. Thus, *u* is bound to the interval [*U*, 1]. Variable *R* describes the depression mechanism and is also increased at every presynaptic spike. Inspired from Markram et al. (1998) the amount depends on the current value of *u*. The strength of depression is controlled via α, which can be any value between 0 and 1. Between spikes *R* decays back to 0 with time constant τ_*R*_. The resulting PSC amplitude is then calculated by the difference of *u_n_* and *R_n_*, scaled by a factor *A*. The PSC decays with time constant τ_*PSC*_.

#### 2.2.2. Circuit implementation

**Figure 3:**
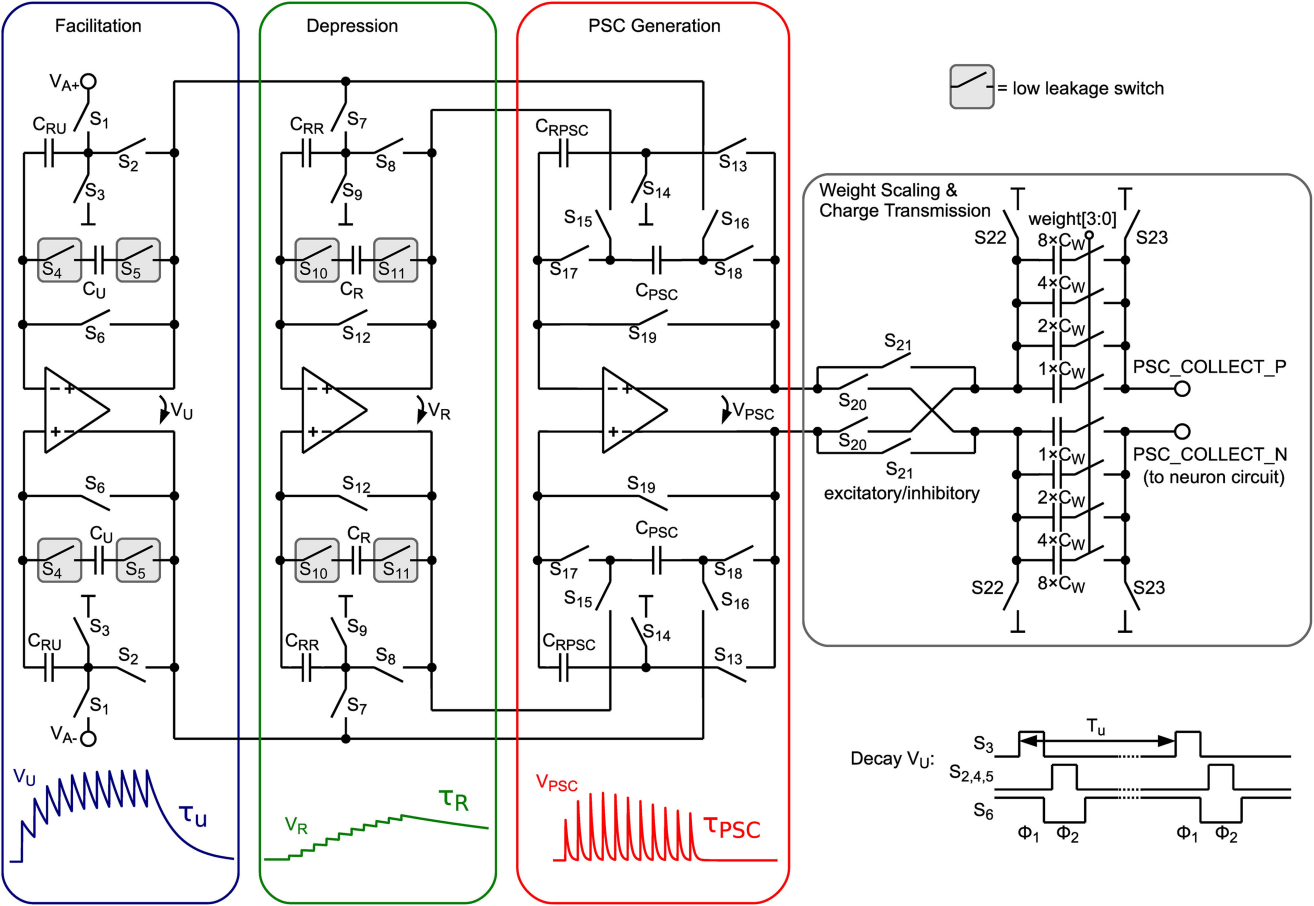
**Schematic of the presynaptic adaptation circuit comprising 3 fully-differential SC leaky integrator circuits**. Capacitors storing the value of the corresponding model variables are encapsulated by dedicated low-leakage switches.

In order to transform the iterative model to continuous-time, the exponential time dependence can be implemented with exponentially decaying voltage traces. These are generated by the circuit shown in Figure 3 for the internal state variables *u*, *R*, and *PSC*, which model facilitation, depression and postsynaptic current trace, respectively. At incoming presynaptic spikes these decay traces are triggered and the resulting PSC amplitude is calculated by the difference of facilitation and depression value as shown in Equation 3. In Figure 3 the circuit schematic is shown comprising three similar parts, for calculating *V_U_*, *V_R_*, and *V_PSC_*.

**Figure 4:**
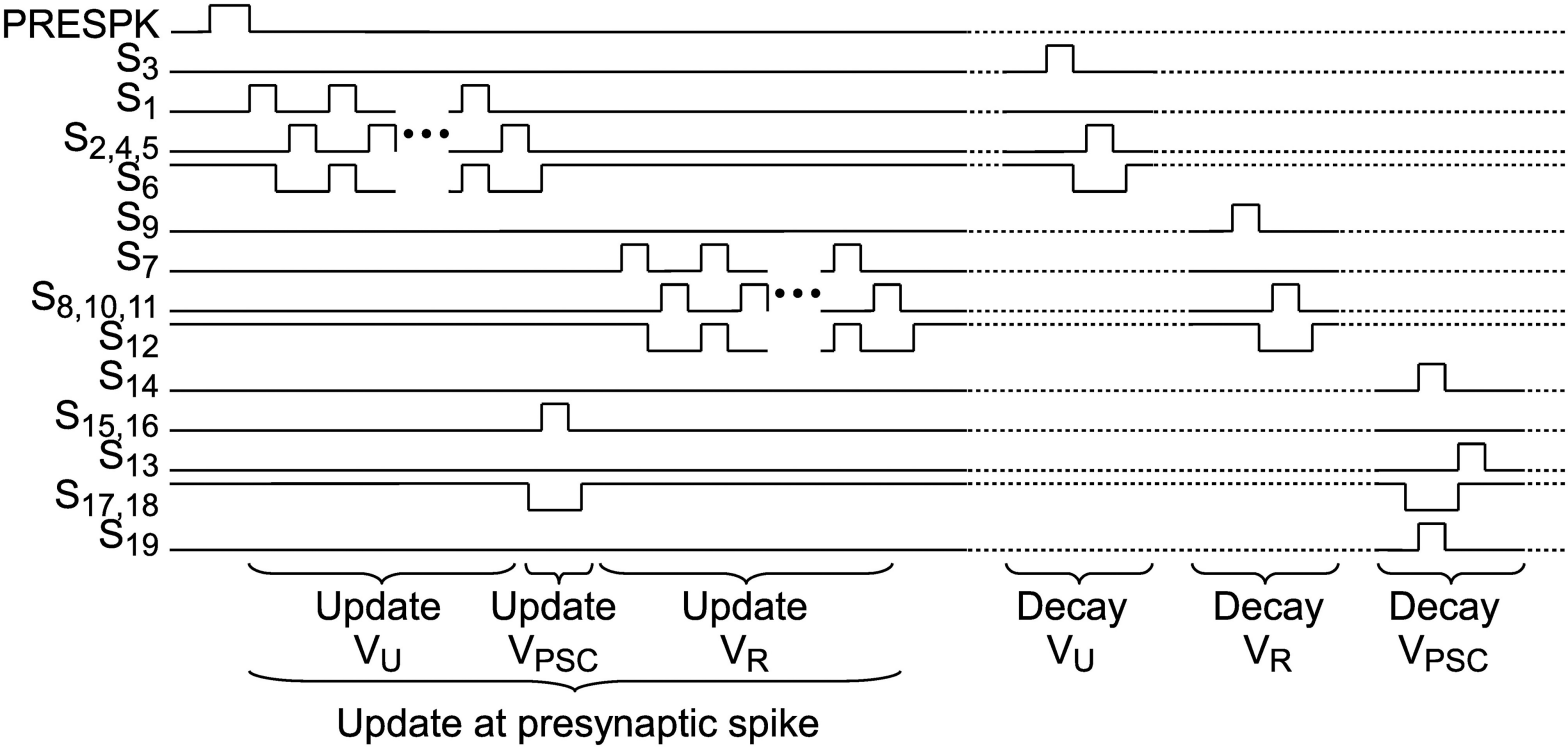
**Switch signals for update at an incoming presynaptic spike and for exponential decays of *V_U_*, *V_R_* and *V_PSC_***. Dotted lines indicate that decay events can occur independently as well as simultaneously.

When a presynaptic spike occurs these voltages are updated by a special switching scheme presented in Figure 4. *V_U_* is increased toward *V_A_*, which represents the global scaling factor *A* in Equation 3. The number of switching events of the *V_U_* update determines the parameter *U*. α is set by the number of switching events of the *V_R_* update. Switches *S*_17_ and *S*_18_ transfer the voltage difference of *V_U_* and *V_R_* to *V_PSC_*.

Between incoming spikes an exponential decay of *V_U_*, *V_R_*, and *V_PSC_* is performed by SC leaky integrator circuits. The working principle will be explained for the facilitation subcircuit and can be applied analogously for depression and PSC generation. On every decay event (see “Decay *V_u_*” in Figure 4) *C_RU_* (5 fF) is discharged in a first switching phase Φ_1_ (see also bottom right of Figure 3). In this period *C_U_* (75 fF), which stores the value of the facilitation variable, is fully decoupled from the circuit. Switching phase Φ_2_ performs a charge equalization on *C_U_* and *C_RU_*. Thus, on every decay event *V_U_* is decreased by a factor $\frac{C_{U}}{C_{U}+C_{RU}}=\frac{15}{16}$. These decay events are repeated with period *T_u_*. With $\frac{15}{16}=\text{exp}(\text{​}-\frac{T_{u}}{\tau_{u}})$ we can easily calculate *T_u_* for a desired decay time constant τ_*u*_:

(4) $$T_{u}=-\tau_{u}\cdot \text{ln}\left(\frac{15}{16}\right)\approx \tau_{u}\cdot 0.0645.$$

Since *T_u_* is derived from a digital counter driven by the system clock, τ_*u*_ is proportional to the counter size and system clock frequency and allows to set time constants ranging from a few milliseconds to about one second. In order to scale the system's overall speed there is a tunable system clock divider, which enables to operate the circuit from biological real-time up to a 100-fold acceleration, keeping all relative timings without the need for adjusting bias voltages.

With the period of the matrix column cycle, the resulting exponentially decaying PSC voltage is sampled on the 4-bit binary-weighted capacitor *C_W_* and transferred to the neuron circuit.

#### 2.2.3. Leakage reduction

**Figure 5:**
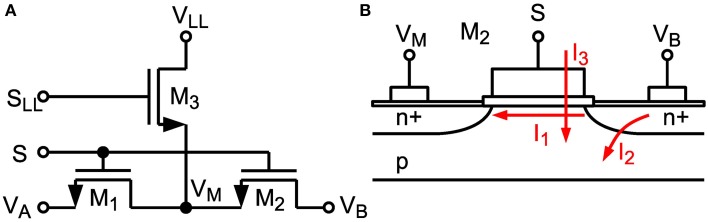
**(A)** Low-leakage switch configuration. **(B)** Cross-section of MOS Transistor M2 with denoted subthreshold leakage (*I*_1_), junction leakage (*I*_2_) and gate leakage (*I*_3_).

The maximum achievable time constant is limited by subthreshold leakage and junction leakage in the switches (see *I*_1_ and *I*_2_, resp. in Figure 5B) (Roy et al., 2003). A dedicated technique similar to Ellguth et al. (2006) and Ishida et al. (2006) has been applied for switches surrounding capacitors *C_U_* and *C_R_* where the switch transistor is split into two transistors (see Figure 5A). If the switch is in off-state the middle node *V_M_* is clamped to a fixed voltage *V_LL_*. Switch signals *S* and *S_LL_* are non-overlapping. With *V_LL_* = 250 mV, which is equal to the common-mode voltage, drain-source voltage of M1 and M2 is kept low, which minimizes subthreshold leakage. Furthermore, the amount of leakage current is independent of the voltage at the other switch terminal. Junction leakage is minimized by minimal sized drain and source terminals. With a reduced voltage swing of about *V_DD_*/2 all switches can be implemented with NMOS transistors only, which keeps leakage currents low and reduces circuit complexity. Especially the concept of isolating capacitors by low-leakage switches makes it possible to reach time constants up to 600 ms, which is the maximum controllable setting in our design, despite using small capacitance values in the 28nm technology node (which naturally has high leakage). This is demonstrated by the measurements in Section 3.2. Thus, we achieve an off-resistance of about 600 ms/75 fF = 8TΩ, which corresponds to a conductance of 125 fS. In contrast to another technique recently proposed by Rovere et al. (2014), which requires two auxiliary low offset opamps, our solution is much more area and power efficient and satisfies our leakage constraints.

#### 2.2.4. Proposed opamp

**Figure 6:**
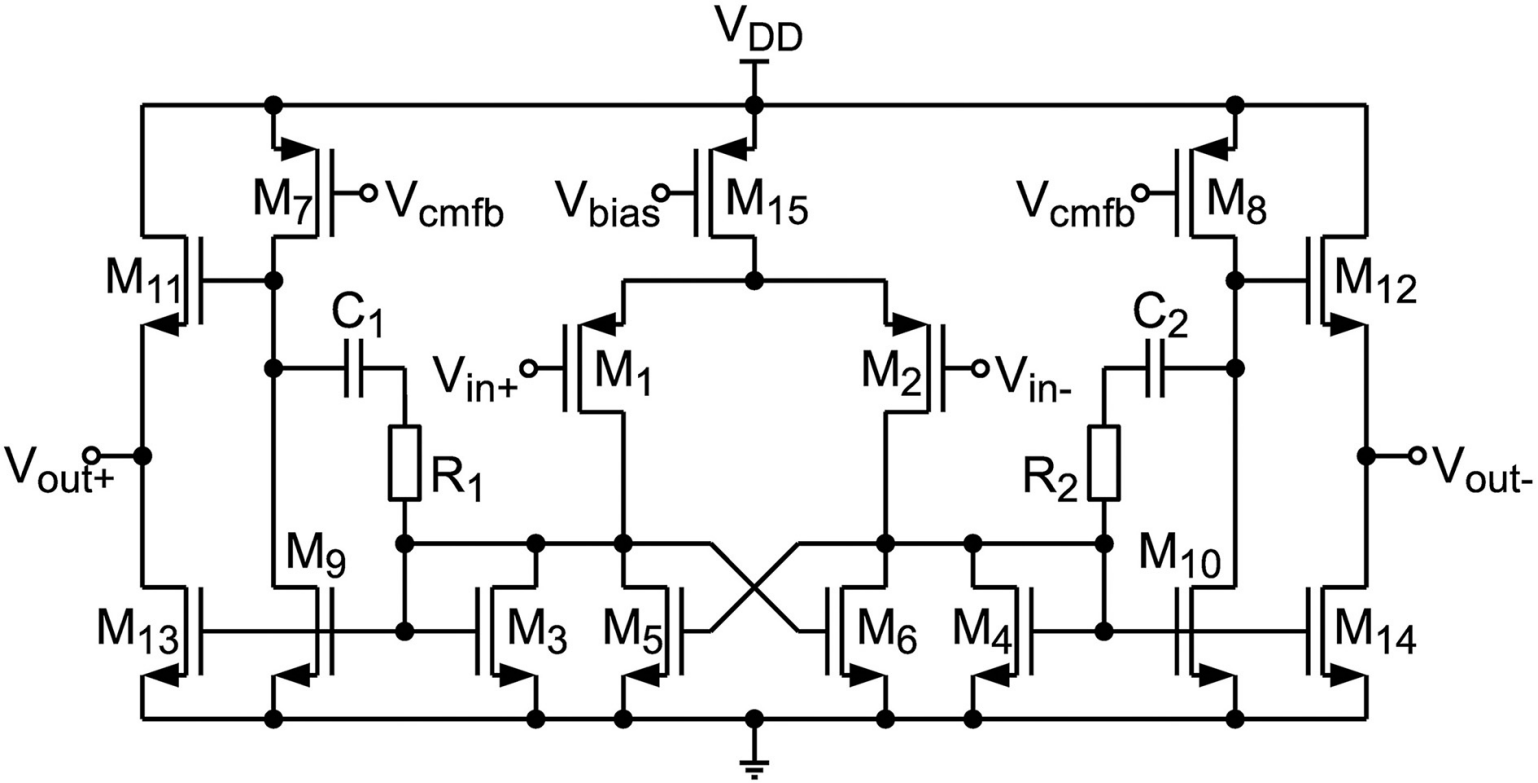
**Proposed opamp circuit used for buffering *V_u_*, *V_R_*, and *V_PSC_***.

For buffering *V_u_*, *V_R_*, and *V_PSC_* a two stage opamp is used (see Figure 6), since transistor stacking is difficult at supply voltages of 1 V. A gain boosting technique similar to Dessouky and Kaiser (2000) has been applied, where the load of the first stage has been split into two cross-coupled transistors (*M*_3_, *M*_5_ and *M*_4_, *M*_6_). By connecting the gates of *M*_5_ and *M*_6_ to the opposite output of the first stage a positive feedback is generated. The common-mode voltage of the first stage is well defined by the diode connected transistors *M*_3_ and *M*_4_ whereas the common-mode voltage of the output stage (*M*_7_–*M*_14_) is controlled by an SC CMFB network. In order to derive stability a classical miller compensation (*C*_1_, *R*_1_, *C*_2_, *R*_2_) has been applied using poly resistors and custom designed metal-oxide-metal capacitors. At the output an NMOS source follower (*M*_11_ – *M*_14_) is connected, which enhances slew rate performance. Thus, the output voltage range is limited to 0–500 mV, which corresponds to the allowed voltage range of the low-leakage switches. The input common mode voltage range is 0–420 mV, which is sufficient for *V_cm_* = 250 mV. The opamp consumes an area of 68μm^2^ and achieves an open-loop gain of 54 dB. It is designed to operate in biological real-time, as well as in a 100-fold accelerated environment. In fast mode the opamp draws 30 μW of power and has a slew rate of 60 V/μs. As the capacitor settling time scales with speed-up, the power consumption in real-time operation can be reduced by a factor of 100, i.e., down to 300 nW.

#### 2.2.5. Offset compensation

Due to the small area occupied by the opamp, which is important for large scale integration, mismatch results in a maximum input offset voltage of about ±16 mV. Nevertheless, this offset can be compensated by a simple auto-zeroing technique (Enz and Temes, 1996). As can be seen in Figure 3, in the sampling phase (Φ_1_) input voltages and common-mode voltages, respectively, are sampled against virtual ground of the opamp (switches *S*_6_, *S*_12_ and *S*_19_ are closed). Since the offset voltage is present at the opamp input at this time, it is also sampled, and thus, canceled out at the output in the second phase (Φ_2_). Despite the existence of more advanced auto-zeroing techniques in the literature, this technique has been chosen, because neither additional capacitors nor additional switching phases are required, reducing area and circuit complexity.

### 2.3. Switched-capacitor implementation of a bistable stochastic synapse

#### 2.3.1. Model

The stop learning model of long-term plasticity has been introduced in Brader et al. (2007), based on earlier work in Fusi et al. (2000). The model represents a synapse with two stable states, potentiated and depressed, whereby the state transition between both stable states is regulated via a continuous internal state X(t) of the synapse. X(t) is influenced by a combination of pre- and postsynaptic activity, namely the presynaptic spike time *t_pre_* and the value of the neuron membrane voltage *V_mem_*(*t*). A presynaptic spike arriving at *t_pre_* reads the instantaneous values *V_mem_*(*t_pre_*) and *C*(*t_pre_*). The conditions for a change in X depend on these instantaneous values in the following way:

(5) $$\begin{array}{l} \begin{array}{llll} \text{X}\to \text{X}+a & if & \{V_{mem}(t_{pre})>\theta_{V} & and \end{array}\\ \;\theta_{up}^{l}<C(t_{pre})<\theta_{up}^{h}\} \end{array}$$

(6) $$\begin{array}{l} \begin{array}{llll} \text{X}\to \text{X}-b & if & \{V_{mem}(t_{pre})\leq \theta_{V} & and \end{array}\\ \;\theta_{down}^{l}<C(t_{pre})<\theta_{down}^{h}\}, \end{array}$$

where a and b are jump sizes and θ_*V*_ is a voltage threshold. In other words, X(t) is increased if *V_mem_*(*t*) is elevated (above θ_*V*_) when the presynaptic spike arrives and decreased when *V_mem_*(*t*) is low at time *t_pre_*. The θ^*l*^_*up*_, θ^*h*^_*up*_, θ^*l*^_*down*_, and θ^*h*^_*down*_ are thresholds on the calcium variable. The calcium variable C(t) is an auxiliary variable (see Brader et al., 2007 for details) that provides a low-pass filter of the postsynaptic spikes. This gives the ability to stop the learning based on thresholded, long-term averages of postsynaptic activity. In the absence of a presynaptic spike or if stop learning is active [i.e., C(t) hits the respective threshold], then X(t) drifts toward one of two stable values:

(7) $$\begin{array}{lll} \frac{d\text{X}}{dt}=\alpha & \;if & \text{X}>\theta_{X} \end{array}$$

(8) $$\begin{array}{lll} \frac{d\text{X}}{dt}=-\beta & if & \text{X}\leq \theta_{X} \end{array}$$

The bistable state of the synapse is determined according to whether X(t) lies above or below the threshold θ_*X*_. Computationally, this model is interesting because through X(t) it can learn a graded response to an input pattern even though the output weight of the synapses is binary. The model also has some biological veracity, being sensitive to pre-post and post-pre spike patterns in a manner similar to the well-known spike time dependent plasticity (Brader et al., 2007).

#### 2.3.2. Circuit implementation

**Figure 7:**
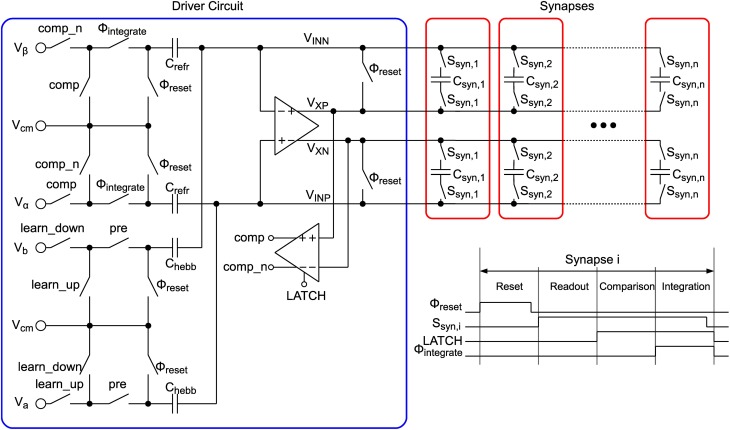
**LTP circuit**.

The circuit schematic shown in Figure 7 replicates the model described in Equations (5–8). In contrast to the circuit presented in (Indiveri et al., 2006) our implementation makes use of SC technique. Thus, the model equations are solved in a time-discrete fashion, which enables the use of low-leakage switches as shown in Section 2.2.3 to achieve very low drift rates α and β. The time-discretization also allows for time multiplexing the single synapse circuits, thus, one driver circuit (see blue box in Figure 7) can drive multiple (in our case 64) synapses (red boxes). Due to the removal of active elements, one synapse circuit can be reduced to only 2 capacitors and 4 low-leakage switches storing the synapse state *X* (cp. Equations 5–8) as a differential voltage. The synapse occupies an area of 3.6 μm × 3.6 μm which is shared equally by the two synapse capacitors with 22 fF each. These are custom-designed metal-oxide-metal capacitors, utilizing an interdigitated fingered layout in the complete 5-layer metal stack with cut-outs on the lower two layers for wiring. The low-leakage switches are located directly below the capacitors. Each synapse can be connected to the driver circuit via switches *S*_*syn,i*_, where *i* indicates the column number in the synapse matrix, and 4 wires *V_INP_*,*V_INN_*,*V_XP_*, and *V_XN_*. The driver circuit is basically an SC integrator, which integrates different voltages *V*_α_, *V*_β_, *V_a_*, and *V_b_* in dependence of synapse state, neuron state and incoming presynaptic spikes onto the synapse capacitors *C*_*syn,i*_. The integrator's opamp is the same as for the presynaptic driver presented in Section 2.2.4. As shown in the timing diagram in the lower right corner of Figure 7, the operation principle can be divided into 4 phases “Reset,” “Readout,” “Comparison” and “Integration” for one synapse. All synapses of one row are cycled through sequentially, whereas all rows are processed in parallel.

In the reset phase an offset compensation of the opamp (cp. Section 2.2.5) is performed, which avoids the integration of a possible offset voltage as well as residual charge on the relatively long wires to the synapses. Therefore, switches annotated with Φ_*reset*_ are closed, which closes a negative unity-gain feedback loop around the opamp. The offset voltage appearing at the opamp input is then stored on capacitors *C_refr_* and *C_hebb_* and can be subtracted in the integration phase.

After reset a readout of the synapse state is performed. Switches *S_syn,i_* of the currently active synapse *i* are closed, which places the synapse capacitors in the feedback path of the opamp. The voltage stored on the capacitors, i.e., the synapse state *X*, is now visible at the opamp output between the differential lines *V_XP_* and *V_XN_*.

When the readout is completed the synapse capacitors stay connected and a comparison of the synapse state with threshold Θ_*X*_ is performed. In the implementation Θ_*X*_ is fixed at 0.5, thus, the comparator (see Section 2.3.3) only has to compare whether *V_XP_* > *V_XN_*. After comparison the result is provided by signals comp and its inverted counterpart comp_n.

In the integration phase the refresh part (see Equations 7, 8) and the hebbian part (Equations 5, 6) of the learning model are performed. In this phase switches annotated with Φ_*integrate*_ are closed. If comp is high then the differential synapse voltage *V_X_* is increased by $\frac{C_{refr}}{C_{syn}}\cdot (V_{\alpha }-V_{cm})$, otherwise it is decreased by $\frac{C_{refr}}{C_{syn}}\cdot (V_{\beta }-V_{cm})$. This results in refresh rates of

(9) $$\alpha =\frac{C_{refr}}{C_{syn}}\cdot \frac{\left(V_{\alpha }-V_{cm}\right)}{\Delta t}$$

and

(10) $$\beta =\frac{C_{refr}}{C_{syn}}\cdot \frac{\left(V_{\beta }-V_{cm}\right)}{\Delta t},$$

where Δ*t* = 0.62 ms, which is the time needed for processing the 64 synapses of a row sequentially (in biological real-time mode).

If a presynaptic input spike arrives, then switch signal pre is high during the integration phase. In dependence of the postsynaptic membrane state Θ_*V*_ signals learn_up and learn_down are set. The neuron circuit providing the membrane state is an SC leaky integrate-and-fire neuron presented in the companion paper Mayr et al. (in press). It is equipped with two comparator circuits for spiking threshold detection and for judging the current membrane state, i.e., the *V_mem_*(*t_pre_*) ≷ θ_*V*_ condition of Equation (5) resp. Equation (6). If *V_mem_*(*t_pre_*) > θ_*V*_, then learn_up is high and learn_down is low (neglecting the “stop learning” mechanism for now). Thus, the upward jump size is calculated by

(11) $$a=\frac{C_{hebb}}{C_{syn}}\cdot (V_{a}-V_{cm}).$$

If *V_mem_*(*t_pre_*) < θ_*V*_, then learn_up is low and learn_down is high, which results in the downward jump size of

(12) $$b=\frac{C_{hebb}}{C_{syn}}\cdot (V_{b}-V_{cm}).$$

In order to reduce the number of control voltages, single-ended input voltages are provided. The resulting common mode offset, caused by this asymmetry, is compensated by the SC CMFB circuit.

The “stop learning” feature described in Section 2.3.1 is handled by setting learn_up resp. learn_down to low using combinational logic (not shown). Therefore, the state of the calcium variable can be calculated externally in an FPGA, where the postsynaptic spike train is filtered by a low pass filter. The low pass filter output is then compared against the stop learning thresholds θ*^l^_up_*, θ*^h^_up_*, θ*^l^_down_*, and θ*^h^_down_* and the two resulting binary signals for enabling learning in the up and down direction, respectively, are transmitted to the driver circuit. As an additional feature for testing we implemented a “learn force” mode where learn_up and learn_down can be set explicitly, similar to keeping the neuron membrane permanently elevated or depressed.

The comp signal, which is provided in the “Comparison” phase states whether the synapse is depressed (LTD) or potentiated (LTP). This binary output is used to scale the PSC generated by the presynaptic adaptation circuit (see “Weight Scaling and Charge Transmission” in Figure 3). Therefore, each synapse has two 4-bit weights for LTP and LTD stored in a RAM (see Figure 1), which is chosen accordingly to the synapse state and transmitted to the weight scaling circuit. The scaling of the PSC is done via binary weighted capacitors, transferring charge to the neuron circuit. Additionally each synapse is selectable excitatory or inhibitory, which inverts the PSC voltage. Thus, inhibitory stop-learning synapses are also possible.

#### 2.3.3. Comparator circuit

**Figure 8:**
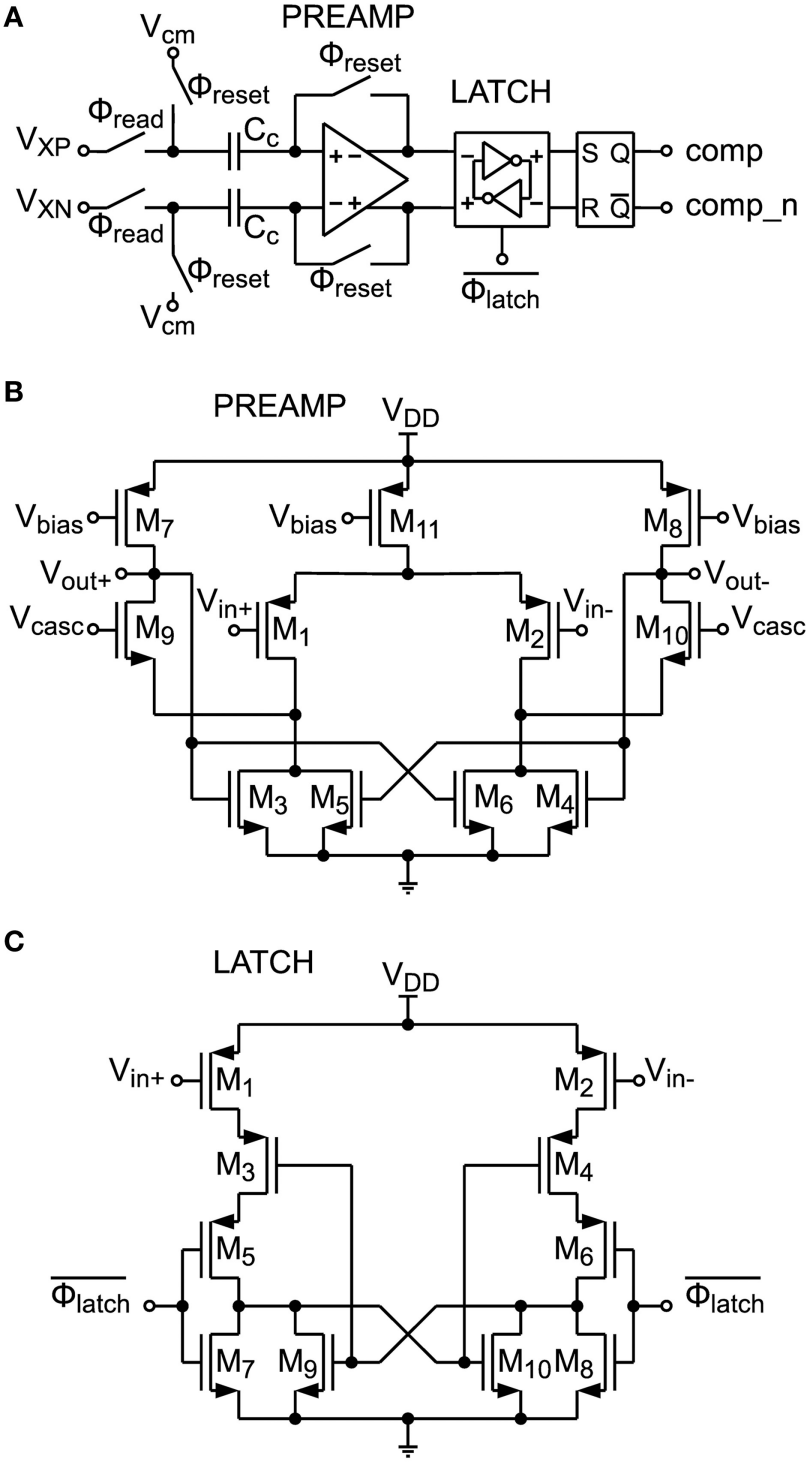
**(A)** Comparator circuit with offset-compensated preamplifier, compensation capacitors *C_c_* and latch circuitry. **(B)** Preamplifier circuit schematic. **(C)** Latch circuit schematic.

A circuit schematic of the comparator shown in Figure 7 is depicted in Figure 8A. It consists of a preamplifier (see Figure 8B), which is inspired by Dessouky and Kaiser (2000) and a simple dynamic latch circuit (Song et al., 1995) shown in Figure 8C. This architecture has been chosen, because the dynamic latch circuit can have a high random offset voltage of up to 20 mV, caused by mismatch. The preamplifier raises the differential signal level to minimize decision errors, caused by this mismatch. The preamplifier is therefore equipped with an offset compensation (compare Section 2.2.5). At the output of the comparator circuit an SR-latch is connected, which stores the result until the next comparison.

### 2.4. Measurement setup and characterization methods

As detailed in Section 2.1, the entire system is ratiometric with respect to the clock frequency. That is, the system clock can be scaled so that the neuromorphic system operates anywhere from biological real time up to a factor 100 faster. As operation at biological real time is the most challenging in circuit terms as well as the most interesting in terms of computation, real-time operation was used for the measurements in this paper. The corresponding clock frequency is 3.3 MHz, generated by a configurable clock divider from the 330 MHz central system clock. At this frequency, the synaptic matrix update period is 0.62 ms (compare Section 2.1).

**Figure 9:**
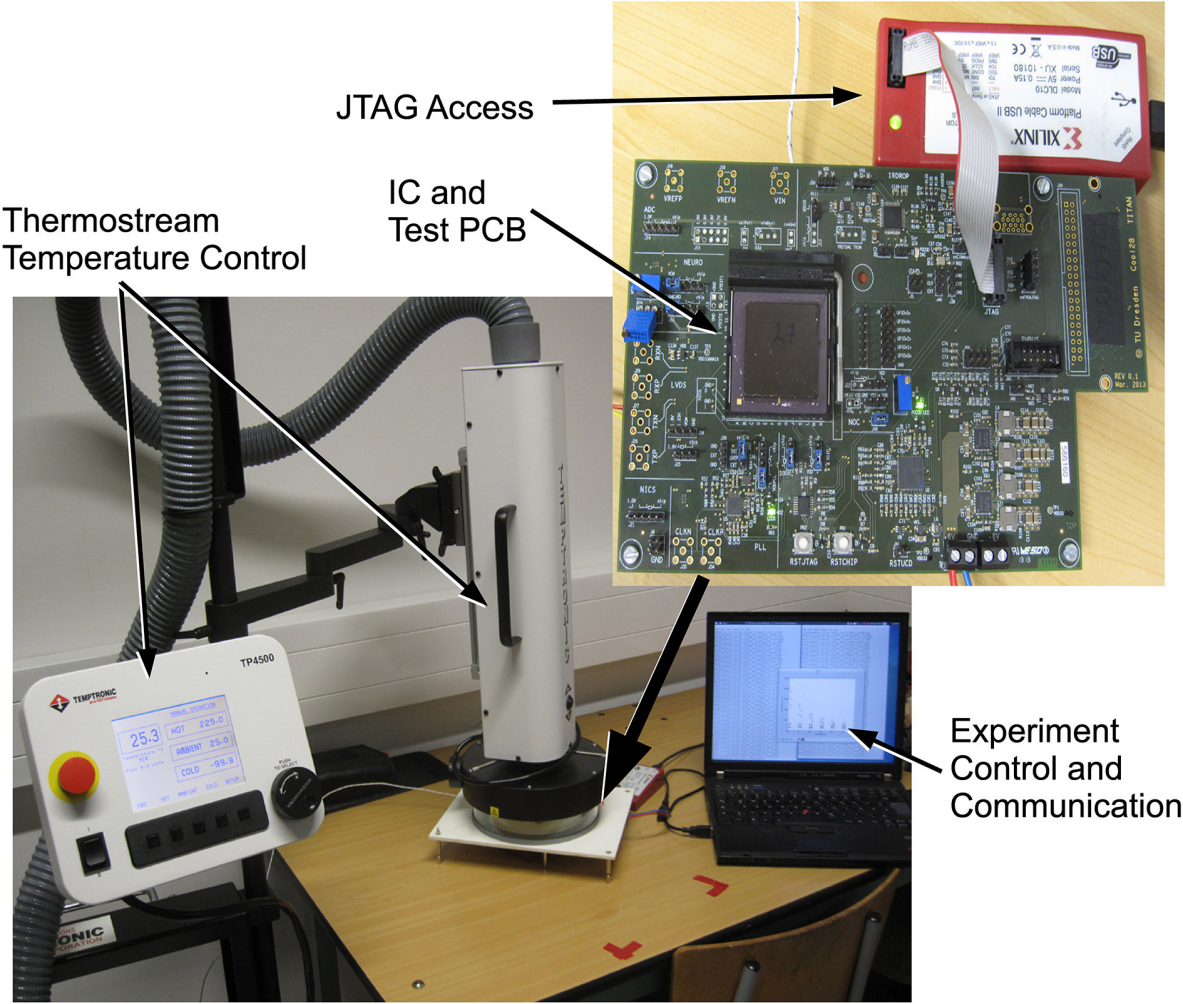
**Setup for measurements with controlled temperature**.

As the different leakage currents of MOS switches are highly temperature dependent, we investigated how well our low-leakage switch technique operates at different temperatures. Thus, the measurements of the presynaptic adaptation are carried out at the temperatures indicated by using the temperature controlled setup shown in Figure 9. The IC package is held at the adjusted temperature with ca. ±2 °C deviation. The output of the presynaptic adaptation can be measured either via tracing the PSC time course from one of the analog test outputs or indirectly by monitoring the spike output of a connected neuron. Directly measuring the PSC voltage via an oscilloscope is well-suited for detailed short-time measurements, which we used to verify correct operation of the circuitry. For reducing noise in this case, the aquired waveform data was averaged over time bins of 0.1–0.3 ms.

Direct oscilloscope measurements are less practical for automatic extraction of a multitude of time constants. For this case, we used the following purely spike-based protocol: The adaptation state is probed by sending an input spike and counting the number of output spikes in reaction. For getting a reasonably strong response, the synaptic weight and the PSC scaling voltage are set to their maximum values. Setting the membrane time constant to a high value as well, the number of output spikes per input spike is approximately linearly dependent on the PSC amplitude. For the measurements, we only activated depression, so that the PSC amplitude of a spike directly resembles the current state of the depression variable. For each time constant measurement, the depression variable is charged by initially applying 10 spikes. Afterwards, the adaptation strength is set to zero, so that the depression variable relaxes back to its resting state. This relaxation is monitored by continuously probing the state with input spikes. From the relaxation time course, the time constant is extracted by calculating the best-fitting (smallest root mean squared error) exponential function, with amplitude and time constant as free parameters. Results are averaged over 10 repetitions.

The measurements of the stop learning synapses are carried out at ambient temperature, i.e., no special measures for chip cooling are taken.

## 3. Results

### 3.1. Basic operation of the presynaptic adaptation

**Figure 10:**
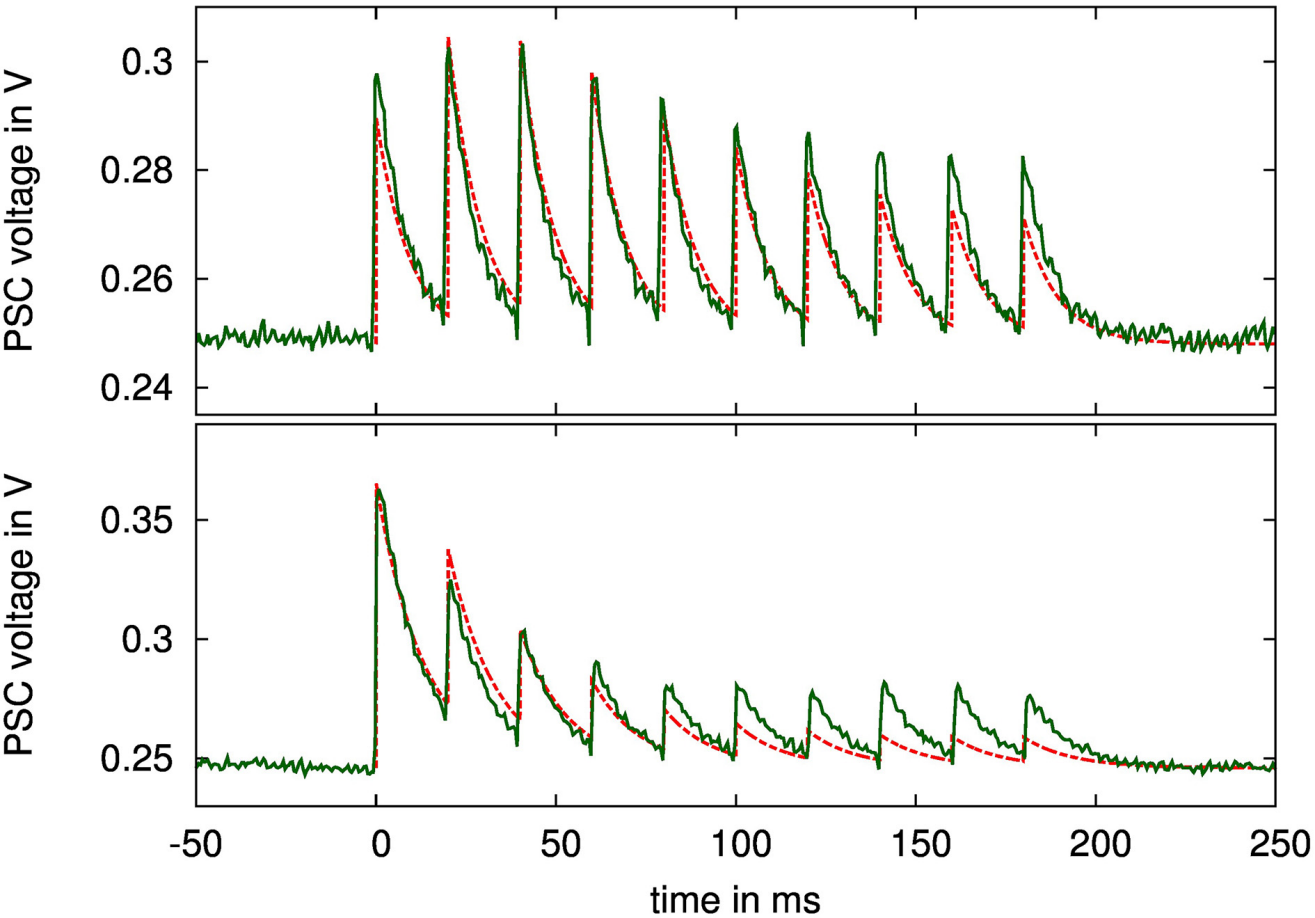
**PSC voltage traces of a simultaneously facilitating and depressing (top), and of a depressing (bottom) synapse when stimulated with 10 spikes at 50 Hz rate**. Configuration parameters: top: τ_*u*_ = 300 ms, τ_*R*_ = 300 ms, τ_PSC_ = 10 ms, *U* = 0.29, α = 0.5, bottom: τ_*u*_ = 10 ms, τ_*R*_ = 490 ms, τ_PSC_ = 13 ms, *U* = 0.96, α = 0.5. The nominal time courses for the PSC voltages with these parameters and fitted amplitudes are drawn as dashed lines.

For evaluating the presynaptic adaptation performance, we stimulated a presynaptic circuit with a regular spike train for two different adaptation types, as shown in Figure 10. We chose a parameter set for combined facilitation and depression to demonstrate correct operation of the circuit as a whole, and a setting for a depressing synapse, where the depression variable dominates the behavior. The latter case is used for assessing the correct reproduction of long time constants in the next section.

Figure 10 also shows ideal time courses for the implemented model with the same parameters and fitted amplitude and offset. The measurements agree well with these nominal curves even without calibrating any parameters. They differ mainly in the adaptation strength, i.e., in the ratio between highest and lowest PSC amplitude, which is smaller in the measured curves. This may be caused by time constants being too small, or by charge injection effects, resulting in voltage offsets during updates of the adaptation variables at incoming spikes.

### 3.2. Characterization of the presynaptic adaptation time constants

**Figure 11:**
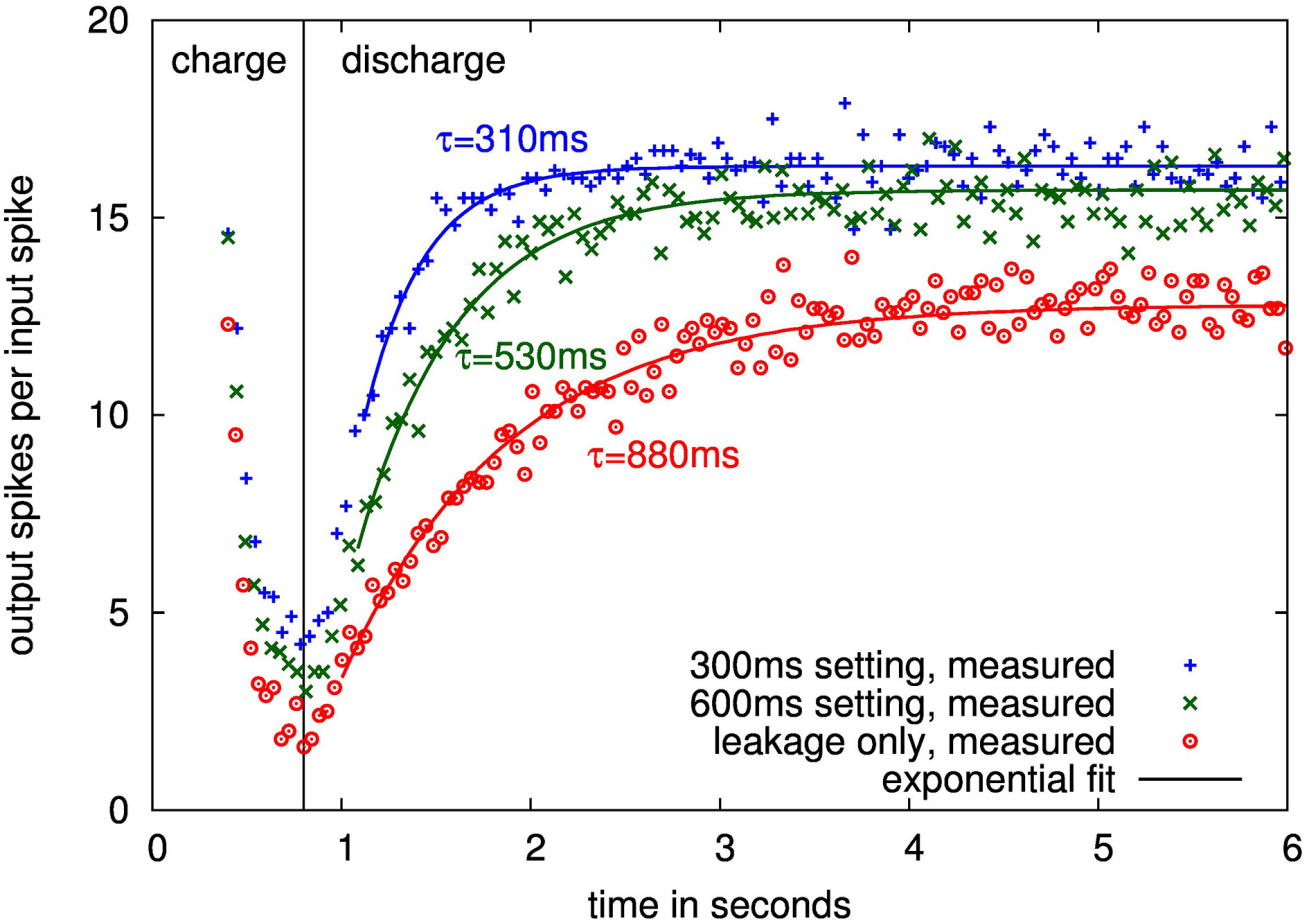
**Measured time courses of input-output gain for one presynaptic adaptation circuit at 40°C with 300 ms, 600 ms and leakage only settings**. Time course until 0.8 s is the charging of the depression, following, the synapse relaxes back to its steady state with the depression time constant.

Figure 11 shows traces over different time constant settings for one presynaptic adaptation circuit. The time course of the depression relaxation for nominal settings as well as with only leakage present can be faithfully fitted by an exponential function, allowing for calculation of the depression time constant.

**Figure 12:**
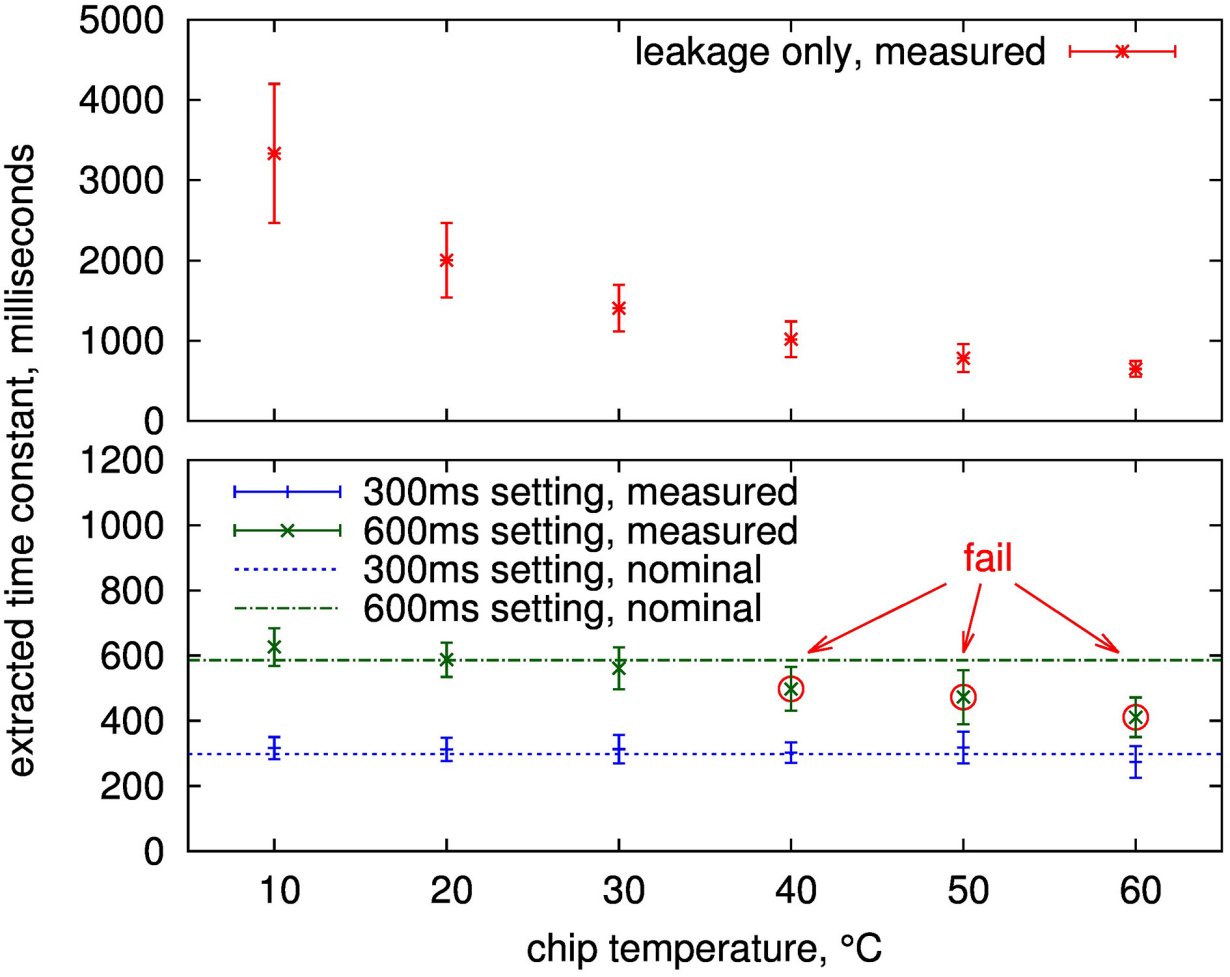
**Mean and standard deviation (error bars) of extracted time constants over 16 presynaptic adaptation circuits of four separate ICs**. Shown is the measured time constant for a setting of infinity (upper part, i.e., the equivalent time constant if just leakage is active) and two configured time constants (nominal 600 and 300ms) for the presynaptic adaptation circuit of Figure 3.

Measured time constants of 16 adaptation circuits from 4 chips are shown in Figure 12. The values are well-controlled in the configurable range up to 300 ms at all temperatures with sigma less than 15% and the mean within 20% of the nominal setting. The same is true for the 600 ms setting up to 30°C. Above that, the leakage influence causes the measured mean to be at least one sigma outside the nominal, which constitutes our fail criterion.

Using the infinite setting for the depression time constant, i.e., there are no decay switching events, this leakage can be measured, see upper plot in Figure 12. As expected, it is highly temperature-dependent. For temperatures of 30°C and below, all measurements are above 1 s, so that time constants up to this value are feasible at room temperature if the controlled leakage, i.e., the switching frequency of the decay process, is further decreased compared to the 600 ms setting. As described in Section 2.2.3, a time constant of 600 ms corresponds to a leakage resistance of 8 TΩ. This value increases to a minimum of 13 TΩ for time constants of 1 s or above. These high resistances demonstrate the effectiveness of the employed leakage reduction techniques.

The measurements show that time constants of several seconds are possible at temperatures below 30°C. As the time constants caused by intrinsic leakage show a larger spread for these temperatures, individual calibration of the switching frequency for the leakage mechanism may be required to still achieve well-controlled time constant values. Nevertheless, for the envisaged time constant range up to 600 ms of the design, the measurements demonstrate correct resemblence of time constant values at room temperature, so that all further measurements were performed without any special measures for temperature control.

### 3.3. Characterization of the bistable stochastic synapse

**Figure 13:**
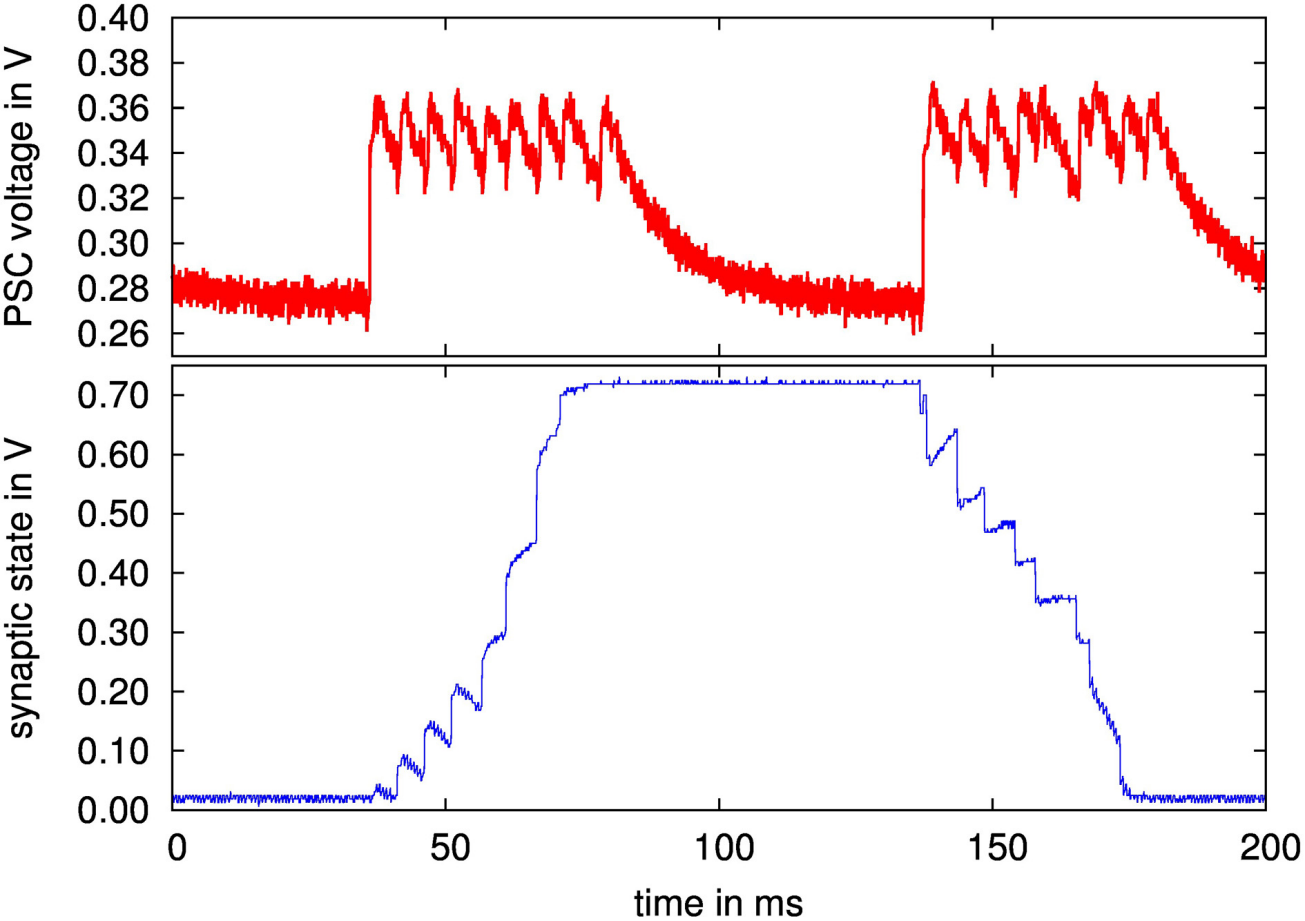
**(Upper diagram) Measured PSC waveform of a 200 Hz presynaptic spike train with 12 pulses; (lower diagram) synapse state of stochastic stop learning synapse, with forced transition from depressed to potentiated state and back**.

In this section, results for the SC implementation of the stop-learning synapse are given. As detailed in Section 2.3.2, a force bit can be set that forces the synapse to transition from potentiated to depressed state or vice versa. That is, Equation 5 resp. Equation 6 are forced to always employ *a* or *b*, similar to setting *V_mem_*(*t*) either to a constant high or low value. A presynaptic spike train of 12 spikes is then applied to the synapse, as shown in the upper diagram of Figure 13.

From the lower diagram of Figure 13, it can be observed that the synapse reaches a stable potentiated state (at ca. 0.7 V) or a depressed state (at 0 V). For the transition at 50 ms, the force bit activates only *a*, forcing the synapse to become potentiated. Conversely, at 150 ms, only b is active, the synapse becomes depressed. Between presynaptic events, the curve shows that α and β draw the synapse back to one of its stable states, according to the synapse state being above or below θ_*X*_ (set at half way between the two stable states, see also Equation 7 resp. 8).

**Figure 14:**
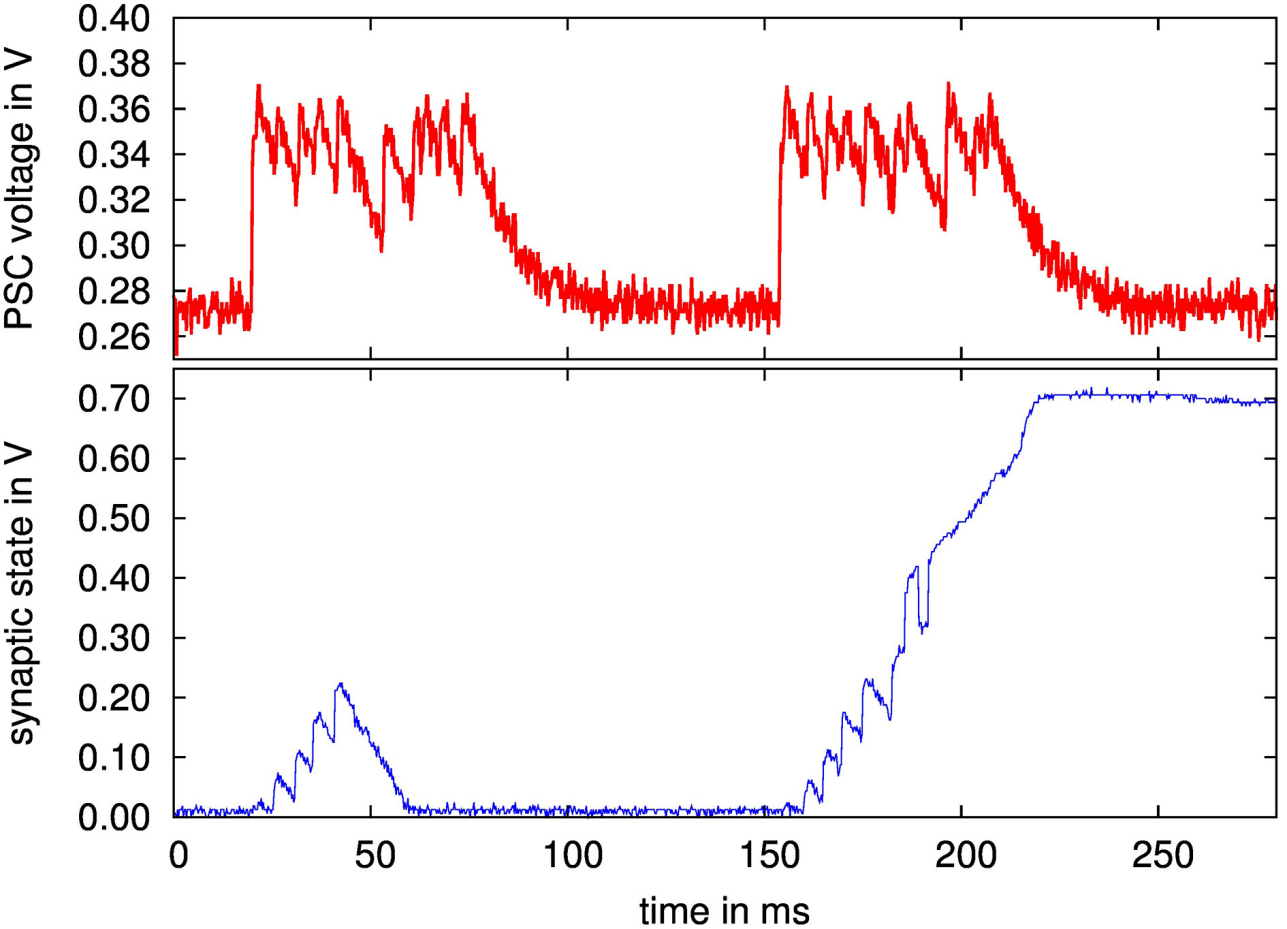
**(Upper diagram) Measured PSC waveform of presynaptic spike train, both packets 12 pulses, 200 Hz; (lower diagram) synapse state of stochastic stop learning synapse, with forced transition from depressed to potentiated state**. The first transition is aborted due to activation of stop learning after 6 pulses, i.e., at a point where the synapse state is not above θ_*X*_ and thus gets drawn back to the depressed state. For the second transition, stop learning is activated after 8 pulses.

To test the stop learning functionality expressed in our implementation by the two stop learning bit flags (see Section 2.3.2), a second experiment is carried out. The packet of 12 presynaptic spikes is split in two parts which are sent immediately after each other, see the corresponding PSC voltage in the upper diagram of Figure 14. Starting from the depressed state, the force bit activates *a*, but after the first part of the presynaptic spike packet, which contains 6 pulses, the stop learning bit for *a* is activated. This causes the last 6 pulses to be discarded in terms of synaptic state modification, i.e., only β is active which draws the synapse back down to the depressed state.

At 150 ms, this experiment is repeated, but the stop learning is activated after 8 pulses. This is sufficient to push the synapse above θ_*X*_, i.e., α becomes active which draws the synapse state to the potentiated state, even though the last 4 presynaptic pulses are again discarded because of the activated stop learning. Thus, overall functionality of the stochastic stop learning synapse is confirmed. In this experiment, the stop learning was set explicitely. As stated in Section 2.3.2, the future backplane for a multi-chip system will compute the Calcium variable externally on an FPGA based on the output spike rates (Brader et al., 2007), setting the stop learning bits dynamically based on the Calcium state.

Please note that we are only showing the internal synaptic state transitions. For the overall network dynamics, the state change means a switch between the 4 bit potentiated and 4 bit depressed weights (compare Section 2.3.2). Thus, while learning induction is in the form of the one bit decision of the original stop learning synapse (Brader et al., 2007), the expression of the synaptic learning can be individual for each synapse, adding significantly to network richness compared to the global settings for potentiated and depressed synapses in other implementations of this plasticity rule (Indiveri et al., 2006). This capability for individual weights could also be exploited for implementations of the Neural Engineering Framework (Eliasmith and Anderson, 2004) on our neuromorphic system. A 4 bit weight resolution plus the capability for setting each synapse excitatory or inhibitory should be sufficient for sophisticated population-based signal processing (Mayr et al., 2014), compare also the results achieved for 58 neurons with 4 bit synaptic weights in Corradi et al. (2014).

### 3.4. Overall results

Table 1 details the major characteristics of the neuromorphic system (Mayr et al., in press). Its power budget is competitive with recent power-optimized digital or analog neuromorphic systems of similar size (Indiveri et al., 2006; Seo et al., 2011). The digital part includes 0.45 mW static power draw which is mainly due to the other components on this test chip, so putting the neuromorphic system on a chip by itself would improve power consumption by about 23% at biological real time operation. The current clocking setup features a constant-frequency PLL (Höppner et al., 2013) and a clock divider, which draw constant power irrespective of the speed up factor. To save power, this could be replaced with a variable-frequency PLL with frequency-dependent power draw (Eisenreich et al., 2009).

**Table 1:** **Characteristics of the presented SC neuromorphic system**.

**Technology**	**Global foundries 28 nm SLP**
Layout area for system	460*430 μm^2^ neuromorphic comp., 600*600 μm^2^ overall (including DAC, RAM, etc.)
Clock frequency	330 MHz (PLL), 3.3 MHz (neuromorphic components)
VDD analog	1.0 V
VDD digital	0.75 V
Power digital	1.1 mW (speed-up 1) to 3.1 mW (speed-up 100)
Power analog (neuromorphic components)	0.38 mW (speed-up 1) to 11.0 mW (speed-up 100)
Power analog (PLL)	0.45 mW
Neuron model	LIAF (Rolls and Deco, 2010)
Presynaptic adaptation	Facilitation and depression (Noack et al., 2012)
Synaptic plasticity	Stochastic synapse with stop learning (Brader et al., 2007)
System characteristics	128 presynaptic adaptation circuits, 8192 stochastic synapses, 64 LIAF neurons

*All figures are for a speed-up of one, i.e., biological real time operation, if not stated otherwise*.

Plasticity models with time constants up to seconds have been shown for this SC implementation in 28 nm. Thus, reliable, controlled behavior fully in keeping with biological real time operation is possible. The efficacy of our chosen method for low-leakage capacitive state holding has been proven, with detailed analysis of the effect of temperature on achievable time constants. The characterization of the presynaptic time constants employs the entire signal pathway of the system (compare Figure 1), showing complete overall functionality.

Table 2 gives a comparison with other current implementations of presynaptic adaptation and/or synaptic plasticity. The synapse area of our implementation is among the lowest, with only the static 1 bit synapse of a digital synaptic array smaller in size. Especially, compared to fully analog implementations of stop learning (Indiveri et al., 2006), the SC approach and agressive scaling for the various capacitances allow an implementation of stop-learning that benefits from the technology shrink. As can be seen from the faithfulness of model replication in SC, this scaling can be achieved without compromising functional richness and accuracy. When accounting for technology node, the area consumption of the presynaptic adaptation is larger than e.g., Bartolozzi and Indiveri (2007) or Schemmel et al. (2010). This is due to the fact that our presynaptic adaptation aims at a very faithful reproduction of the model of Markram et al. (1998), necessitating complex, multi-stage computational circuits (see Figure 3). Specifically, our implementation is the only one offering concurrently operating facilitation and depression.

**Table 2:** **Comparison of the presented short- and long-term plasticity circuits with other implementations from literature**.

**Ref**.	**Techn**.	**System area**	**Synapse area**	**Number of synapses**	**Synapse functionality**	**Pre-synapse area**	**Number of presynapses**	**Presynapse functionality**
Seo et al., 2011; Merolla et al., 2011	45 nm	4.2 mm^2^	1.6 μm^2^	262 k	1-bit static synapses, Set externally	–	–	Not implemented
Park et al., 2014	90 nm	16 mm^2^	15 μm^2^	262 k	Log-domain conductance-based synapse, no plasticity	–	–	Not implemented
Mitra et al., 2006; Bartolozzi and Indiveri, 2007	350 nm	12 mm^2^	1200 μm^2^	8192	Stop learning	1360 μm^2^	NA	Short-term depression
Schemmel et al., 2010; Schemmel, personal communication	180 nm	50 mm^2^	150 μm^2^	115 k	STDP	84 μm^2^	14 k	Either short-term depression or facilitation
This work	28 nm	0.36 mm^2^	13 μm^2^	8192	Stop learning	432 μm^2^	128	Concurrent short-term depression and facilitation

The shown architecture always connects an input via synapses to all neurons, corresponding to an all-to-all connectivity. This is the same architecture as used for example in memristive crossbar arrays Alibart et al. (2012); Mayr et al. (2012). The main advantage of this architecture in our design is that it allows to implement all parts of the synapse circuit that depend on the input only once per synapse row. This significantly reduces circuit area, reducing the synapse circuit to an analog storage element in our design. The efficiency gain comes at the price of reduced flexibility concerning connection topologies. All-to-all and comparable connection structures are well-suited, whereas sparse connectivity results in a high number of unused synapses in the matrix, making the architecture less efficient in this case, even when optimizing the mapping of networks to the hardware architecture Mayr et al. (2007); Galluppi et al. (2012). To improve the efficiency, i.e., the fraction of utilized synapses, also for low connection densities, more presynaptic input circuits than synapse rows can be implemented, while synapses are made to choose between several inputs (Noack et al., 2010; Schemmel et al., 2010). This would only slightly increase the complexity of the individual synapse circuits, while greatly increasing the flexibility of the architecture (Noack et al., 2010).

## 4. Discussion

### 4.1. Plasticity models

Results show faithful implementation of the chosen short-term plasticity model (Markram et al., 1998). The detailed reproduction of this model endows the neuromorphic system with a corresponding rich behavioral repertoire, which could be employed for e.g., reproduction of population dynamics in cultured neurons (Masquelier and Deco, 2013) or simulation of short-term memory (Rolls et al., 2013).

The long-term plasticity rule is also reproduced well, opening up a host of information-theoretic applications, such as studies of memory retention, information content or classification performance of a network (Brader et al., 2007). Other flavors of long-term plasticity rules could also be supported by our neuromorphic system. For instance, the faithful reproduction of neuronal waveforms evident in Figure 10 and their excellent configurability in terms of the time window (Figure 12) could also be employed for a plasticity rule based on neuron and synapse waveforms such as (Mayr et al., 2010), which aims at the replication of a wide range of biological plasticity experiments (Mayr and Partzsch, 2010).

### 4.2. Switched-capacitor neuromorphics

Dating back to Carver Mead, subthreshold CMOS has been the mainstay of neuromorphic circuit design, as it offers the advantage of low power consumption, ion-channel like behavior in CMOS devices and currents small enough to reach biological real time operation. However, such a fully analog implementation suffers from mismatch and leakage currents which are increasingly prevalent in deep submicron processes. In addition, the channel-to-transistor design philosophy means that this type of neuromorphic circuit consists largely of handcrafted circuits that depend crucially on the performance of each single transistor. Thus, porting a design between technology nodes essentially means a completely new design.

Switched-capacitor neuromorphic circuits move from this device level philosophy to a building block approach, i.e., the required model behavior is achieved with a combination of standard building blocks. SC is used as a mathematical framework to directly translate state-driven models to a mixed-signal realization. This keeps the neuronal states analog for biological veracity, while achieving significantly easier technology porting, as the circuit consists solely of standard building blocks such as amplifiers, switches and charge addition/subtraction. Representation of analog states at block level also eases implementation in deep submicron, as this takes advantage of the available device count for improved signal fidelity, while relying less on the characteristics of individual transistors. This building block approach allows agressive scaling of the active analog components, while the digital part of the SC circuits naturally scales with the technology node. Overall scaling is ultimately limited compared to a purely digital system by the largely invariant capacitor sizes, but is still significantly better than conventional, more device- and analog-centric neuromorphic approaches. As shown, this approach has enabled our SC system to deliver the same computational density as a purely digital neuromorphic system in a deep-submicron technology (Seo et al., 2011), while its power budget is on par with subthreshold circuits (Indiveri et al., 2006). When combined with deep submicron pixel cells (Henker et al., 2007), a sophisticated visual processing pyramid could be implemented (König et al., 2002; Serrano-Gotarredona et al., 2009).

While SC makes neuromorphic circuits possible in principle in deep submicron, one major challenge is still the leakage currents. The leakage completely precludes subthreshold circuits, but it also affects the stored states of capacitors in SC technique, especially for the timescales necessary for biological real time operation. As shown, we have solved this general challenge for SC neuromorphic circuits with our low leakage switch architecture, reaching controllable time constants >100 ms at ambient temperature.

### 4.3. Nanoscale CMOS and novel devices

Novel nanoscale devices, such as memristors, offer the possibility of very high density neuromorphic synaptic matrices (Alibart et al., 2012; Shuai et al., 2013). However, they need corresponding high-density neuronal driver circuits in CMOS. Moving neuromorphic circuits to deep-submicron technologies as outlined in this paper would provide this capability, i.e., very low footprint neuron driver and receiver circuits that generate analog waveforms for memristor synaptic matrices (Mayr et al., 2012).

### Conflict of interest statement

The authors declare that the research was conducted in the absence of any commercial or financial relationships that could be construed as a potential conflict of interest.
